# Keeping nurses engaged during COVID-19: An i-deal perspective

**DOI:** 10.4102/sajip.v48i0.1971

**Published:** 2022-10-28

**Authors:** Precious Ngobeni, Nelesh Dhanpat

**Affiliations:** 1 Department of Industrial Psychology and People Management, College of Business and Economics, University of Johannesburg, Johannesburg, South Africa

**Keywords:** COVID-19, nurses, i-deals, work engagement, customised work arrangements, private hospitals

## Abstract

**Orientation:**

The coronavirus disease 2019 (COVID-19) pandemic has impacted all job sectors. Arguably, the hardest hit were healthcare institutions. Nurses are at the front line, and it is known that the pandemic added pressure to the way nurses performed their duties. Their working schedules became more complex, including longer hours, as nurses dealt with high rates of COVID-19 cases while still dealing with other healthcare issues.

**Research purpose:**

The study aimed to establish the relationship between idiosyncratic deals (i-deals) and work engagement of nurses. The study focused on these three types of i-deals - task, flexibility and career. It investigated which i-deals best predict work engagement among nurses.

**Motivation for the study:**

There is a need to understand the work arrangements of nurses during the pandemic through i-deals. Although research on idiosyncratic deals has become popular in international research, there is scant research within the South African context.

**Research approach/design and method:**

The sample consisted of 220 nurses working in three private hospitals in Gauteng, South Africa. Inferential statistics and regression analysis were used to achieve the research objectives.

**Main findings:**

The study’s findings revealed a correlation between the three types of i-deals and work engagement. However, only task and flexibility i-deals predicted work engagement. The COVID-19 pandemic added pressure to the healthcare industry and to nurses’ challenges. The pandemic highlighted the importance of having an engaged nursing workforce. Thus, recommendations and suggestions for nurses, nursing managers and human resource managers are provided.

**Practical/managerial implications:**

The concept of i-deals is a reasonably new phenomenon within HR practices, and there is no empirical research within the South African context.

**Contribution/value-add:**

The study adds value by providing insight into customised work arrangements, from an i-deal perspective, during a much appropriate time and urgently needed for nurses.

## Introduction

The coronavirus disease 2019 (COVID-19) pandemic exacerbated healthcare workers’ skills and mental and emotional capacities (Fernandez et al., 2020), thus contributing to the existing challenges of the healthcare system globally. In the context of South Africa, COVID-19 gripped the already struggling healthcare system, mainly affecting the frontline workers involved in fighting the pandemic (Robertson et al., 2020). It was noted that psychological distress such as anxiety and depression increased among healthcare workers. Nurses were noted to be the most involved frontline workers in fighting the outbreak of COVID-19 (Kim et al., 2021).

In this regard, nurses were required to work longer hours, under increased restrictions and often unfamiliar settings, while continuously wearing personal protective equipment (PPE) (Dramowski et al., 2020). Physical exhaustion, separation from family, and losing loved ones and colleagues resulted in nurses’ evaluating their career paths. The pressures of the pandemic deepened the challenges confronting nurses within their working environment (Dramowski et al., 2020). Nurses are expected to deliver quality healthcare, save lives and achieve set organisational goals, despite the challenges they experience (Shamian et al., 2016). They are thus expected to comply with robust standards, policies and procedures that may place patients’ needs and emotions before their own (Beukes & Botha, 2013). At times, these standardised procedures may hinder a nurse from performing well, thus prompting nurses to enter into an arrangement with their supervisor. In addition, the stress created in this profession is likely to negatively affect nurses’ work engagement and quality of life (Karatepe & Avci, 2017). Nurses’ vital role in the health industry must be accompanied by attractive and motivational retention strategies (Dhanpat et al., 2019).

It will be interesting to understand customised work arrangements through idiosyncratic deals (i-deals) in the nursing context. Individualised work customisation occurs through negotiation between an employer and employee for different working arrangements compared to other employees doing a similar job; this is known as an i-deal (Bal, 2017). Highly skilled employees have realised that they offer specialised skills and expertise that the organisation values and may use this advantage to bargain for individual tasks and working environments (Liao et al., 2016). The concept of i-deals and work engagement has been under-researched, and limited empirical research on i-deals exists in a South African context. Therefore, the primary aim of the research is to establish the relationship between i-deals and nurses’ work engagement during the COVID-19 pandemic. The research also sets out to assess which i-deals best predict work engagement of nurses.

## Literature review

The employment relationship is constantly changing as the world of business changes because of economic, technological, social and political factors (Birtch et al., 2016). The workplace has become less structured, and there has been an increase in more flexible forms of employment. Organisations have seen a decrease in collective bargaining, an increase in individually negotiated agreements, and informal working arrangements are becoming more significant in the workplace (Hornung & Rousseau, 2017). Highly skilled employees are becoming more vocal in negotiating work arrangements that differ from those of their colleagues (Villajos et al., 2019). Specialised working arrangements, such as i-deals, retain and attract talented individuals (Laulié & Tekleab, 2016). When employees are permitted to participate in the decision-making process by management, it can minimise employee turnover intention (Christian et al., 2011). When decision-making involves both employees and employers, it generally shows that the opinions and contributions of employees are recognised and rewarded (Belete, 2018).

### Theoretical underpinnings of idiosyncratic deals and work engagement

The premise of social exchange theory (SET) is that relationships are formed over time when there is mutual commitment, trust, and loyalty among employers, employees and colleagues (Birtch et al., 2016). Social exchange theory can be traced back to the 1920s, bridging different disciplines such as organisational behaviour, social psychology and sociology (Wu & Lee, 2017). Social exchange theory can be defined as voluntary interactions that are co-dependent on the actions of another person (Cropanzano et al., 2017). The concept of i-deals and work engagement is underpinned by SET (Liao et al., 2016).

The exchange made usually benefits the employer–employee relationship. Employers will accept i-deals to reciprocate an employee’s hard work and value their contribution to the organisation. However for an employee, the acceptance of the i-deal by their manager leads to positive attitudes and behaviours that will increase performance and provide a competitive advantage to the organisation (Hornung et al., 2014). A mutually beneficial relationship between employer and employee is positively related to the successful negotiation of i-deals, such as preferred job activities (Hornung et al., 2010; Rosen et al., 2013), career development opportunities (Hornung et al., 2014) and flexibility with work scheduling (Liao et al., 2016; Rosen et al., 2013).

The exchange requires a collaborative transaction; something has to be given and returned (Harden et al., 2018). In other words, if a person supplies a benefit, the receiving party should respond with an equal benefit. The reciprocal exchange rule encourages cooperation and reduces risk. Decreasing organisational politics and organisational injustice ultimately influences an employee’s intention to stay within the organisation (Harden et al., 2018). Social exchange theory may therefore be linked with work engagement, as when employees are more engaged in a work environment, they feel psychologically safe and will behave positively, despite work challenges (Cook et al., 2013). Beneficial and fair transactions between an employer and employee result in a stronger social exchange relationship. The relationship will produce efficient and effective work behaviour and positive employee attitudes. Social exchange relationships grow when employers ensure that employees are provided with growth opportunities, training and development, a conducive working environment and suitable rewards (Parzefall & Salin, 2010).

### Idiosyncratic deals

The concept of i-deals is recent and has emerged under the interest of organisational sciences (Rousseau et al., 2018). Idiosyncratic deals can be defined as special terms of employment negotiated between employees (present or prospective) and their employers (present or prospective) that benefit both parties’ needs (Bal et al., 2013). It can also be explained as valuable employees seeking to negotiate individualised working conditions with their employers outside of traditional practices generally available to employees (Ng & Feldman, 2010). An employee may use i-deals to balance work and personal life (Rousseau, 2001). They can be diverse and unique for employees who seek them, and they tend to be negotiated in different ways (Rousseau & Greenberg, 2006). While others exist, only the following three will be discussed for the purpose of this study.

#### Career idiosyncratic deals

Career i-deals assist employees to negotiate conditions that help them accomplish longer-term set developmental goals (Hornung et al., 2010), such as being involved in important committees or training and growth opportunities. Career i-deals may also be referred to as development i-deals. These i-deals are usually negotiated after an employee is hired (Ng & Feldman, 2010). The successful negotiation of career i-deals is related to improved individual performance, work engagement, motivation and commitment to the organisation’s goals and vision. Career i-deals may put an employee in a strategically advantageous position over their colleagues, in that they are considered first for career opportunities and significant or highly sought-after assignments (Rousseau, 2001). Thus, this type of i-deal tends to be negotiated by highly skilled or ‘star’ performers who also have a good relationship with their employer.

#### Task idiosyncratic deals

Task i-deals involve negotiated changes to the job content and description that makes the employee’s work more intrinsically interesting or pleasing (Ng & Lucianetti, 2016). Through such i-deals, employees request preferred duties or responsibilities related to their intrinsic interests or skills. Previous research suggests that task i-deals motivate employees to take greater initiative on the job and affect their work engagement (Hornung et al., 2018). Task and development i-deals can also be used as tools when workers have issues with their performance. For example, providing training or adjustments in job responsibilities can help poor performers to improve (Ng & Feldman, 2010).

#### Flexibility idiosyncratic deals

Flexibility i-deals allow employees to work according to their desired schedule or at their choice of location (Hornung et al., 2010). Such i-deals tend to be negotiated with trusted employees, especially in organisations that have flexible policies. However, employees who negotiate such i-deals will need the permission of their employer to work according to their preferred schedule or location (Ng & Lucianetti, 2016). Employees who negotiate flexibility i-deals may need to make special and consistent arrangements to ensure that work is done, contributions are seen and all parties benefit (Hornung et al., 2010). For example, such efforts can include constant updates to supervisors and colleagues regarding milestones met, goals achieved and contributions made (Lee & Hui, 2011).

### The need for idiosyncratic deals among nurses

Nurses are considered the backbone of any healthcare system (Staufenbiel & König, 2010). Their role is vital in ensuring that procedures and policies are successfully implemented, and quality healthcare services are provided. In addition, nurses are at the forefront of interacting with patients daily (Staufenbiel & König, 2010). Although the nursing profession can be satisfying and rewarding, it is not without its challenges that can make nurses less efficient in rendering quality healthcare services (Ronnie, 2019). When nurses do not focus at work, they may make mistakes, resulting in slow recoveries or even unnecessary deaths (Jabarkhil et al., 2021). The challenges faced by nurses are perhaps the basic motivators for them to leave their profession or negotiate for individualised working conditions (Huo et al., 2014).

Idiosyncratic deals represent the expectations and requests that highly skilled nurses require from their employer to become more engaged in the nursing environment (Huo et al., 2014). Thus, nurses will negotiate for individualised working arrangements that differ from those of their co-workers, as skilled nurses know their value and the health institution’s obligation towardsthem. Situations such as nurses’ being forced to cancel leave, work extra hours, multitask and perform non-nursing work such as inventory-taking may lead to the negotiation of an i-deal. This i-deal should result in desirable working conditions that benefit the health institution. It is paramount that all health institutions ensure that their nursing staff are skilled, motivated, committed and supported, despite the rigorous demands of the healthcare system (Huo et al., 2014).

A study by Bal and Jansen (2015) showed that 80% of severe violent altercations reported were caused by interactions with patients. However, often incidents are not reported by nurses, for fear of losing their jobs (Bal & Jansen, 2015). Nurses can negotiate an i-deal with their shift supervisor to work in a different department, for example, if they are not happy in their current department (Vidyarthi et al., 2016). Violence is just one example of an issue that may hinder a nurse from performing their job effectively. Health institutions need to be aware of the myriad challenges that result in staff shortages and nurse turnover (Bal & Jansen, 2015). Moreso, the COVID-19 pandemic has brought upon various difficulties for the nursing profession, and along with challenges that plagued the profession such as long working hours (Allande-Cussó et al., 2021), and psychological distress symptoms associated with depression and anxiety (Hu et al., 2020).

When management is aware of the challenges and reasons for job dissatisfaction among nurses, retention strategies will be created. These strategies should not be standardised for all nurses, but individual strategies should be created to retain and keep highly skilled nurses engaged (Ho & Kong, 2015). There is need to consider how such symptoms and stress levels can be reduced in order to protect nurses from the impact of the pandemic (Allande-Cussó et al., 2021). It is important to improve the well-being and engagement levels of nurses. It is known that the establishment of i-deals presents special conditions of employment attributed towards customised work arrangements (Rousseau, 2005). Recent studies have explored the various factors that influenced psychological well-being and perceived stress of health care workers during the COVID-19 pandemic (Arden & Chilcot, 2020; Lai et al., 2020). Although such perceived factors are understood, little is known about keeping nurses engaged during the COVID-19 pandemic. This study set out to establish this through the association of i-deals of nurses. To our knowledge there is no research of such within the South African context. Gaining insight into such will be beneficial in sustaining engaged nurses through the negotiation of i-deals.

### Work engagement

Work engagement is recognised as an essential element in determining the extent of employee performance, effectiveness, innovation, commitment and competitive advantage in organisations across the globe (Aguenza & Som, 2012; Othman, 2020). The phenomenon of work engagement can be defined as a state of mind that is positive, fulfilling and characterised by vigour, dedication and absorption (Rameshkumar, 2020; Schaufeli & Salanova, 2007). Work engagement can also be defined as having a positive attitude towards an organisation’s values, environment, workers and business. In addition, work engagement can be defined as an individual’s willingness to go beyond simple satisfaction of one’s work arrangements and committing to assisting an employer to reach their goals (Schaufeli & Salanova, 2007). Engaged employees are, however, not superhuman, as they will also feel tired after a long day of hard work (Bakker et al., 2008). However, their tiredness in these cases can be linked to satisfaction, a positive state of mind and a feeling of self-accomplishment. In addition, engaged employees thrive under pressure, as an increased workload provides fulfilment in knowing that they can achieve and complete work (Bakker & Oerlemans, 2019).

#### Implications of engaged employees

An organisation cannot be sustained for an extended period without engaged employees (Reijseger et al., 2017). Employees who are engaged increase efficiency and generate a favourable business environment. Employee engagement creates positive attitudes among employees about their jobs. For example, when employees are engaged, they do their job with passion and excitement. In addition, engagement creates a workforce that aligns the employees’ activities with the organisation’s strategy, goals and objectives (Reijseger et al., 2017). Previous authors noted that engagement is a vital determinant for high levels of employee performance. For example, engaged employees will become more motivated and determined when performing their job, despite the challenges they may encounter (Ilies et al., 2017).

In this regard, engaged employees will ensure that customers have a positive experience by delivering high-quality products and services (Ilies et al., 2017). When customers are satisfied with what the organisation provides them, they become loyal and refer the service or products to other people, thus increasing profits of the organisation. Quality and efficiency should be a habit for an engaged workforce; this boosts an organisation’s competitive edge in the market in which they operate (O’Bryan & Casey, 2017). Engaged employees exert discretionary efforts by ensuring that they work harder to reach desired goals and objectives, for example, employees who come to work earlier than required to complete work or assist with extra work (Reijseger et al., 2017).

#### Consequences of disengaged employees

An organisation’s reputation begins and ends with its employees, who are at the forefront in interacting with various stakeholders crucial to the success of the organisation (Aguenza & Som, 2012). Therefore, disengaged employees cripple an organisation’s success and have the power to decrease its profits. Disengagement results in unhealthy work relationships between the disengaged employee and their employer and co-workers (Reijseger et al., 2017). As a result, there will be lack of commitment, loyalty and respect when the employee is performing their job.

Employees who are not happy will spread their disengagement to otherwise engaged employees or prospective employees (Reijseger et al., 2017). A disengaged employee is likely to make more errors than an engaged employee when performing their job. In some contexts, these may even result in accidents or safety hazards, which are detrimental to the sustainability of the business (O’Bryan & Casey, 2017).

### Work engagement among nurses

A social service occupation such as nursing needs engaged workers to operate successfully (Cao et al., 2019). Employee engagement can be viewed as a constructive indicator of commitment and loyalty, while some employees use it to repay the organisation for the monetary value (salary and benefits) they receive (Hamid & Shah, 2017). A high level of work engagement among nurses is important for health institutions, as it contributes to satisfied patients and a successful organisation. For example, engaged employees are increasingly more inclined to be more focused, provide solutions to problems and prioritise the organisation’s interests (Cao et al., 2019).

In South Africa, nurses have a vital role to play in the healthcare system’s sustainability, growth and effectiveness (Beukes & Botha, 2013). It is of significance to understand the organisational and personnel variables that motivate nurses to stay in or leave a healthcare institution (Ziedelis, 2019). Individuals who have the perception that their work is just a job and a source of financial gain will only be engaged for the benefits the job provides. However, individuals who perceive their job as a calling, a source of meaning and career advancement will commit more time, energy and loyalty to their work activities (Ziedelis, 2019). A previous study on nurses in South African hospitals found that meaning of work and seeing work as a calling predicted work engagement and organisational commitment (Beukes & Botha, 2013).

In this regard, nurses who viewed their work as a calling were more engaged and committed to their employing institution than nurses who viewed their work as simply a job (Beukes & Botha, 2013).

Work engagement relies on meaningful work. Health institutions should thus strive to provide a working environment that allows an employee to be engaged (Mauno et al., 2016). South Africa is one of the few developing countries that may boast of moderate to excellent healthcare institutions (Bakker, 2018). The nation’s healthcare system can be categorised into public and private institutions. The majority of South Africans cannot afford private healthcare institutions and thus depend on access to public hospitals. Whether employed in a private or public institution, nurses are at the forefront of attending to patients’ needs (Bakker, 2018). Previous studies found that nurses working in a private hospital were more engaged than nurses working in public hospitals (Shahpouri et al., 2016). Studies of nurses indicated that nurses also suffered from psychological distress during the COVID-19 pandemic Some nurses preferred to emigrate because of better benefits and working environment in other countries (Labrague, 2020). It is not only the responsibility of nurses to ensure that they are engaged when performing their work, but it is also the responsibility of management to ensure that they create an environment conducive to high levels of engagement (Bakker, 2018). Moreso, it was established that during the COVID-19 pandemic, nurses experienced psychological distress (Preti et al., 2020). Although this was the case, in another study it revealed that nurses experienced high levels of work engagement (Gómez-Salgado et al., 2021).

## Method

### Research design

A positivist philosophy was used in this study. The study emphasised the positivist focus on rigorous scientific procedures to result in clear data and facts not influenced by human interpretation or prejudice (Saunders et al., 2009). This study followed a quantitative research approach. A quantitative research approach was used as there is a need to explore i-deals from an empirical perspective. A cross-sectional design was employed for this study.

### Data collection

Prior to the data analysis the authors obtained ethical approval from the Department of Industrial Psychology and People Management Ethics Committee from the University of Johannesburg before the study commenced (ethical clearance code IPPM-2019-370[M]). After that, the authors applied for approval at private healthcare providers to research their healthcare institutions in Gauteng. The authors first had to obtain permission from the hospital managers of each healthcare institution before receiving final approval from the research committee of the healthcare providers. The research committee provided final research approval through email. The approval letter was also emailed to the hospital managers to discuss the procedure used to collect data, especially considering the COVID-19 protocols that required social distancing and the use of protective attire. Furthermore, the authors were provided specified times to distribute the questionnaire. The authors agreed with the clinical department that the unit managers would distribute hard-copy questionnaires. Once nurses had completed the questionnaires, they deposited them into a sealed box situated in the clinical department office. The authors collected the sealed boxes from the clinical offices. No staff member had access to the questionnaires within the locked collection box. This ensured the integrity of the data collection procedure.

### Sampling and participants

A non-probability convenience sampling strategy was selected for this study. The sampling criteria for the study included enrolled auxiliary nurses, enrolled nurses, and registered nurses in private hospitals. Because of time constraints and access to the hospitals, 220 responses were received, with a response rate of 48.88%. Access was given to three hospitals in the Johannesburg region. A total of 450 questionnaires were distributed. Lenth (2001) acknowledged that a limited amount of data and observations are noted because of resource constraints. Considering this was the height of the pandemic in South Africa, access to such a sample (nurses) was challenging.

The sample comprised of 15% men (*n =* 33) and 85% women (*n =* 187). The ages ranged from 24 to 64 years, and the median age was 38 years. The majority of participants identified as belonging to the black race group (*n* = 183, 83.56%). Only 8.18% (*n* = 18) were employed part-time. Most respondents were employed as registered nurses (*n* = 120, 54.55%), and most respondents had been employed for between 5 and 10 years (*n* = 163, 74.1%).

### Measuring instruments

The study used pre-established surveys. Questionnaires are versatile because they can cover many subjects or issues or be simple and focus on one aspect or area. Questionnaires are mainly considered because they effectively collect rich data, are inexpensive and provide simple interpretation (Saunders et al., 2012). To obtain demographical information about the respondents, a biographical questionnaire was used. The aspects assessed sample-specific information relating to the participants, such as sector of employment, marital status, employment status, category of nursing, gender, ethnicity, years of service, working experience, age and highest qualification. A scale developed by Hornung et al. (2014) measured i-deals. A shortened (nine-item) version of the Utrecht Work Engagement Scale (UWES-9), developed by Schaufeli et al. (2002), measured work engagement.

#### Idiosyncratic deals scale

The I-Deals Scale is a nine-item measure of dimensions task i-deals, career i-deals and flexibility i-deals. The items are scored on a 5-point Likert scale ranging from 1 (*not at all*) to 5 (*to a very great extent*) and include statements such as ‘A work schedule customised to my personal needs’ and ‘Jobs tasks that fit my personal interests’. Hornung et al. (2014) reported acceptable Cronbach’s alpha coefficients for the three types of i-deals, namely 0.80 for task i-deals, 0.88 for career i-deals, and 0.78 for flexibility i-deals. The current study obtained overall acceptable alphas of 0.90 for task i-deals, 0.88 for career i-deals and 0.88 for flexibility i-deals.

#### Utrecht work engagement scale-9

The UWES-9 is a nine-item measure of dimensions vigour, dedication, and absorption were used to measure engagement. The items are scored on a 7-point frequency scale ranging from 0 (*never*) to 6 (*always*) and include statements such as ‘I feel happy when I am working intensely’. In their study, Patience et al. (2020) achieved an overall Cronbach’s alpha of 0.88 for this measure of work engagement. The current study achieved an overall acceptable alpha of 0.90.

### Data analysis

The authors analysed the data using Statistical Package for Social Sciences (SPSS) (version 25). A statistician assisted with the analysis of the data. A report was generated to analyse the data’s relevance, reliability, validity and accuracy (Rouder et al., 2016). Statistical analysis techniques included descriptive statistics, exploratory factor analysis (EFA), inferential statistics analysis and multiple regression analysis. The study used the EFA to identify a small set of factors that can represent the relationship among a group of related variables in this study, namely, i-deals and work engagement. The Kaiser–Meyer–Olkin (KMO) measure of sampling adequacy should achieve a value of 0.60 or above to verify that data are suitable for factor analysis. At the same time, Bartlett’s test of sphericity should achieve a significant value of 0.05 or smaller. A rotation was performed; the study used the oblimin rotation method with Kaiser normalisation.

Pearson correlation was the inferential statistics tool used, with the values it produces represented by *r* (Pallant, 2011). The study used standard multiple regression analysis to separately assess the predictability of the independent variable, namely, i-deals (task, flexibility and career), on the dependent variables, which was work engagement. Furthermore, standard regression analysis was used to establish the best predictor of i-deals on work engagement (Pallant, 2011).

### Ethical considerations

Ethical clearance to conduct this study was obtained from the University of Johannesburg, Department of Industrial Psychology and People Management Research Ethics Committee (reference number: IPPM- 2020-406[M]). The consent form provided details to participants about anonymity and confidentiality of the information gathered from them. No personal information was requested or collected from the participants. The clinical supervisor also informed participants of their right to withdraw; if the participants wished to stop participating or felt uncomfortable filling in the questionnaire, they were under no obligation to do so. Therefore, consent from respondents was established, based on their voluntary agreement and submitting of the questionnaire. To ensure anonymity, participants were required to place a cross or a tick, to acknowledge their consent. The participants were asked to bring the completed questionnaires to the clinical office and submit them via the sealed box at their convenience. The questionnaires were stored in a sealed container by the clinical manager to ensure privacy.

## Results

### Descriptive analysis

The descriptive analysis for i-deals (task, career and flexibility) is presented in Table 1. The overall mean score achieved for task i-deals was 3.45. This suggests that nurses perceived that they had successfully negotiated against the tasks they performed, to some extent. Based on the frequency analysis, more than half of the nurses (57.3%) perceived that they had successfully negotiated for job tasks aligned to their personal strengths and talents. An overall mean score of 3.40 was obtained for career i-deals. This suggests that nurses negotiated more for career i-deals closer to their professional advancements than personal goals. Based on the frequency analysis, more than half (52.7%) of the employees had negotiated career options that fit their own personal motives. An overall mean score of 3.02 was obtained for flexibility i-deals. Based on the frequency analysis, half (50%) of employees perceived to have negotiated for flexibility options in terms of their work schedule. All i-deals achieved an overall acceptable alpha above 0.70, indicative of internal consistency. The overall mean score achieved for work engagement was 4.13. This suggests that nurses were generally engaged when performing their job. Based on the frequency analysis, a majority (51.8%) of employees were delighted about the job that they performed. Work engagement achieved an overall acceptable alpha of 0.90, indicative of internal consistency.

**TABLE 1:** Descriptive analysis of idiosyncratic deals and work engagement.

Factors	*M*	Skew.	Kurt.	Lower bound	Upper bound	SD	Var.	*α*
Task i-deals	3.45	−0.50	−0.61	3.30	3.60	1.13	1.28	0.90
Career i-deals	3.40	−0.40	−0.67	3.25	3.56	1.16	1.35	0.88
Flexibility i-deals	3.02	−0.09	−1.02	2.86	3.18	1.20	1.44	0.88
Work engagement	4.13	−0.25	−0.70	3.98	4.28	1.13	1.28	0.90

i-deals, idiosyncratic deals; Skew., skewness; Kurt., kurtosis; Var., variance; α, Cronbach’s alpha; SD, standard deviation.

### Factor analysis

The nine items in the I-Deals Scale were subjected to principal axis factoring. The correlation matrix presented communalities above 0.30. The scale achieved a KMO value of 0.84, exceeding the value of 0.60 recommended by Pallant (2011). Bartlett’s test of sphericity reached statistical significance and thus supported the factorability of the correlation mix. Principal axis factoring revealed the presence of three components with an eigenvalue exceeding 1, explaining 52.42%, 11.98% and 8.88% of the total variance. Oblimin rotation with Kaiser normalisation was performed. A three-factor solution was supported upon inspection of the scree plot.

The items of the UWES-9 were subjected to principal axis factoring. The correlation matrix presented communalities above 0.30. The KMO value was 0.89, exceeding the recommended value of 0.60 (Pallant, 2011). Bartlett’s test of sphericity reached statistical significance, thus supporting the factorability of the correlation mix. Principal axis factoring revealed the presence of two components with an eigenvalue exceeding 1, explaining 56.83% and 11.93% of the total variance.

Pallant (2011) suggested that a factor loading above 0.40 is strong. There were two factors extracted through principal axis factoring, and all items had loadings above 0.40. Catell’s (1966) scree plot confirmed a two-factor solution, with eigenvalues of 5.12 and 1.1. Although a two-factor solution was established, previous South African studies have consistently reported one factor of work engagement (De Crom & Rothmann, 2018; Rothmann, 2017). Hence, it was decided to retain only one factor.

### Inferential statistics

A Pearson product–moment correlation was conducted to establish the relationship between the i-deals dimensions (independent variable) and work engagement (dependent variable). The correlations between the following can be noted in Table 2: Task i-deals were positively related to work engagement, *r*(218) = 0.428, *p* ≤ 0.01, suggesting a large effect. Career i-deals were positively related to work engagement, *r*(218) = 0.362, *p* ≤ 0.01, suggesting a medium effect. Flexibility i-deals were positively related to work engagement, *r*(218) = 0.345, *p* ≤ 0.01, suggesting a medium effect. The variables all presented a positive relationship with work engagement, thus suggesting that employees who successfully negotiated for task, career and flexibility i-deals were engaged in their work.

**TABLE 2:** Correlation analysis for idiosyncratic deals and work engagement.

Dimensions	1	2	3	4
1. Task i-ideals	-	-	-	-
2. Career i-deals	0.543**	-	-	-
3. Flexibility i-deals	0.563**	0.472**	-	-
4. Work engagement	0.428**	0.362**	0.345**	-

i-deals, idiosyncratic deals.

** , Correlation is significant at the 0.01 level (two-tailed).

### Regression analysis of idiosyncratic deals predicting work engagement

Standard multiple regression analysis (see Table 3) was used to investigate which type of i-deals (i.e., task, career and flexibility; independent variable) best predicted work engagement (dependent variable). The prerequisites of ensuring that there was no violation of multicollinearity, singularity, outliers, normality, linearity and homoscedasticity were tested before analysis. A significant regression equation was found, *F*(3.216) = 19.74, *p* < 0.000. The total variance of the dependent variable was 21.5%. As a result, this study has interpreted the beta values with confidence (on the recommendation of Pallant, 2011, who suggested that when the variance inflation factor is greater than 10, multicollinearity problems exist within the data analysed). Table 3 shows that task i-deals predicted the largest variance for work engagement (β = 0.278, *p* < 0.001). The second largest variance for work engagement was from career i-deals (β = 0.157, *p* < 0.035). Flexibility i-deals, however, did not significantly predict work engagement, as the *p* value was greater than 0.05.

**TABLE 3:** Regression analysis of idiosyncratic deals predicting work engagement.

Model	Unstandardised coefficients		Standardised coefficients	*T*	Sig.	*R*	*R* ^2^	Adjusted *R*^2^	SEB of the estimate	Collinearity statistics	
	*B*	SE	β							Tolerance	VIF
Constant	2.318	0.246	-	9.421	0.000	0.464	0.215	0.204	1.01	-	-
Task i-deals	0.279	0.079	0.278	3.529	0.001	-	-	-	-	0.584	1.711
Career i-deals	0.153	0.072	0.157	2.117	0.035	-	-	-	-	0.665	1.504
Flexibility i-deals	0.108	0.071	0.115	1.527	0.128	-	-	-	-	0.644	1.552

i-deals, idiosyncratic deals; *B,* Unstandardised coefficient and constant for the linear regression equation; SEB, standard error of *B*; β, standard regression coefficient; VIF, variance inflation factor; SE, standard error.

Predictor (constant): Task i-deals, Career i-deals, flexibility i-deals. Dependent variable: Work engagement.

## Discussion

The objective of the study was to establish a relationship between i-deals and work engagement of nurses. More so, we are aware that employees have experienced various changes in their work due to the pandemic (Dhanpat et al., 2022). Hence, it was essential to understand how employees customise their work by negotiating i-deals. When observing the results of the descriptive statistics it was established that nurses preferred to negotiate for task i-deals that they were good at, allowing them to perform their jobs better. A job task is unlikely to be performed well if the needs and requests of the jobholder are not considered (Rofcanin et al., 2018). More so, nurses preferred to negotiate for career i-deals linked to their own set career goals rather than organisational objectives. Health institutions must support and guide nurses with career development opportunities through creating personalised, unit- and department-specific professional development plans and programmes (Hisel, 2020). Nurses negotiated flexibility concerning working hours outside of the normal standardised working hours. This is unsurprising, due to the stressful working environment that challenges nurses, for example, working longer hours because of workload and shortage of human capital (Wan et al., 2018a). After all, nurses are only human, and it is therefore expected that they would negotiate for a flexibility i-deal to cope with their environment. Nurses were adequately engaged. Therefore, the above suggests that despite nurses’ daily challenges, they are still proud to be part of the profession. Despite the aim for perfection and standardised practices in the nursing environment, work engagement among nurses needs to be considered (Labonté et al., 2015). Work engagement is a vital tool, especially within social service professions such as nursing (Bakker, 2018).

### The relationship between idiosyncratic deals and work engagement

The results of the correlation analysis showed that task i-deals were positively related to work engagement. Thus, nurses’ work engagement in private hospitals may be influenced by the brokering of task i-deals with their supervisors. Owing to the high expectation among patients regarding quality healthcare within private hospitals, nurses have to perform beyond their job descriptions to meet these expectations and will thus make arrangements with their line managers to continue meeting patient expectations and work needs (Shams et al., 2021). Task i-deals had the largest variance in predicting work engagement.

During the COVID-19 pandemic, the findings of Schoberer et al. (2022) suggested that nurses were assigned additional duties. Zhan et al. (2020) confirmed that the main stress-promoting variables for nurses were to accomplish more work under time constraints with limited resources. A Danish study also revealed that the nursing managers performed duties that they did not originally perform before the COVID-19 pandemic (Hølge-Hazelton et al., 2021). The increase in task and work responsibilities will result in nurses negotiating on their responsibilities because of the COVID-19 pandemic. In doing so, this will assist in increasing nurses’ engagement levels. This was confirmed by Zhang et al. (2021) indicating that nurses’ work engagement increased during the pandemic due to the self-dedication nature of the nursing profession.

Employers’ engagement with employees in discussions around task i-deals provides essential insight into employee job preferences and interests (Ng & Feldman, 2010). It also provides an advantage to employers by creating reward and retention structures that will be suitable for employees they value and wish to keep in the organisation. Management will have an idea and knowledge around the job activities that keep nurses engaged. Employers can use task i-deals to balance job tasks with personal needs, thus improving work engagement (Zhang et al., 2021). Task i-deals can create new precedents for work characteristics, thus positively contributing to employee engagement among nurses (Zhang et al., 2021).

The results showed that career i-deals were positively related to work engagement. This suggested that nurses become more engaged when career i-deals are successfully negotiated, as they are keen on the development opportunities agreed upon by their managers. The nursing profession provides nurses with career advancement opportunities, hence the need to negotiate for career i-deals. As this study investigated, the negotiation of career i-deals may be an additional factor contributing to work engagement. The encouragement of professional development by nursing management results in a positive work environment, promotions, employee engagement and increased nurse retention (Hisel, 2020). The regression analysis showed that career i-deals had the second largest variance, thus predicting work engagement. Career i-deals benefit an individual and impact the organisation and co-workers (Lee & Chung, 2019). This is because career i-deals can also be used as an opportunity for knowledge and skills sharing. A culture that promotes professional development results in a continuously updated organisation with the information and necessary skills to keep up with global changes (Magbity et al., 2020). Nursing management is urged to create cultures that negotiate career i-deals and promote professional development (Magbity et al., 2020).

A moderate relationship exists between flexibility i-deals and work engagement. This suggests that when nurses successfully negotiate for flexibility i-deals, their level of work engagement increases. Nurses can negotiate for flexible i-deals such as work schedules and personal and financial arrangements (Ho & Tekleab, 2016). For example, a nurse may negotiate for a salary advance with their supervisor when in financial distress. The study results suggest that nurses require a form of flexibility within their work environment to become engaged at work (Wan et al., 2018). However, this study discovered no significant predication between flexibility i-deals and work engagement. The world is constantly changing, and the healthcare sector is also a party to the change (Labrague & De Los Santos, 2021). Therefore, nursing managers would be oblivious not to realise that the needs and expectations of nurses have changed. Alhough more research needs to be done on links between flexibility i-deals and work engagement, it is evident that even the nursing environment requires a certain level of individualised work arrangements, to cater to the needs of nurses and contribute to work engagement positively (Labrague & De Los Santos, 2021).

### Limitations of the study

Research is never void of limitations, and some limitations have been identified in the current study. COVID‑19 hindered data collection in that there were restrictions in the way data had to be collected in hospitals, which caused delays in data collection and restrictions on the reach of nurses. This included the distances that had to be kept between the authors and participants, and nurses in the COVID-19 wards could only answer the questionnaires without the researcher being present. These factors could lead to questions being answered without clarity on some of the study’s variables. Lastly, the study was only conducted within a specific group of hospitals representing the private sector. Therefore, the results should be interpreted with caution, as the study cannot be generalised to other industries or professions. Nevertheless, the authors perceive the data collected to be noteworthy, contributing to human resource literature and highlighting a need for further exploration by future researchers.

### Implications for management

**FIGURE 1:**
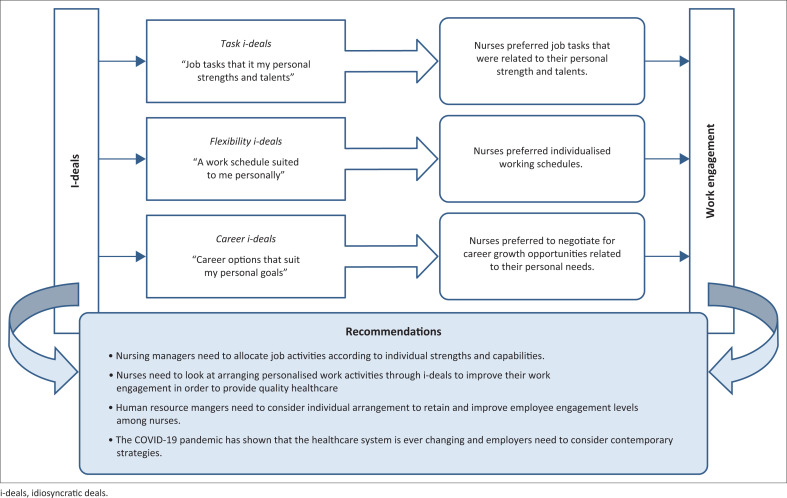
A holistic framework of the relationship between idiosyncratic deals and work engagement.

A practical implication of this study is that the nursing industry is already facing challenges with a shortage of nurses. Figure 1 presents a holistic framework concerning i-deals and its influence on work engagement of nurses. New solutions are needed to retain current nurses and recruit graduate nurses. Therefore, HR managers can use i-deals as a talent attraction and management tool for potential employees and as a retention strategy for current employees. Bearing relevant legislation in mind, the study provides theoretical implications regarding customised employment contracts instead of standard contracts provided to all employees and customised work arrangements. For example, working hours can become flexible, taking into consideration operational requirements. HR managers can look at transformational practices through i-deals and can include this in their balanced scorecard under organisational culture or performance management. Nurses are core professionals of the healthcare system, and the COVID-19 pandemic only served to attest to this. Nursing managers need engaged employees to improve organisational culture and provide quality patient care. It is important to also recognise that offering of flexibility and customised work arrangements is dependent on managerial culture, filtering down from top management and the frame of reference of nursing and hospital management.

### Contribution of the study

The study contributes to the literature of i-deals, and work engagement. Insight was provided into which i-deals best predict work engagement among nurses. Secondly, there is limited research regarding the concept of i-deals in a South African context in general (see Dhanpat & Legoabe, 2022), while research into these variables collectively has not been done in the nursing profession specifically. This suggests that i-deals should be considered as a tool to improve work engagement levels. Hornung et al. (2018) noted that nursing managers need better retention strategies to compete with other organisations and retain skilled nurses. Thus, the study also challenged traditional human resource practices by looking into contemporary practices such as i-deals to adapt to changing environments. Notably, the study comes at a time when customised work arrangements are much needed because of the implications brought about by the COVID-19 pandemic.

## Conclusion

The inferential statistics of the study demonstrated that there was a relationship between i-deals and work engagement, thus showing that nurses most likely negotiated i-deals during the pandemic to cope with their stressful work environment. The regression analysis revealed that two out of the three types of i-deals predicted work engagement; namely, task and career i-deals. The study further showed that task i-deals had a large variance and effect towards work engagement among nurses in private institutions. Therefore, nurses negotiated for job tasks that were related to their strengths. This also showed that nurses preferred to negotiate for i-deals that were related to their work activities to improve their work engagement.
